# Obesity cardiomyopathy could contribute to sudden cardiac death: a Japanese epidemiological morphological study

**DOI:** 10.1186/s12933-024-02456-z

**Published:** 2024-10-24

**Authors:** Ryo Kaimori, Haruto Nishida, Mari Tamura, Kohji Kuroki, Kumi Murata, Kazuhiro Kawamura, Shinjiro Mori, Tsutomu Daa

**Affiliations:** 1 Department of Forensic Medicine, Faculty of Medicine, Oita University, 1-1 Idaigaoka, Hasama-machi, Yufu, Oita 879-5593 Japan; 2 Department of Diagnostic Pathology, Faculty of Medicine, Oita University, 1-1 Idaigaoka, Hasama-machi, Yufu, Oita 879-5593 Japan

**Keywords:** Obesity, Metabolic syndrome, Obesity cardiomyopathy, Cardiomyopathy, Sudden cardiac death, Pathology, Autopsy

## Abstract

**Background:**

We aimed to clarify the existence and pathological features of obesity cardiomyopathy (OCM) in Japan using our series of autopsy cases.

**Methods:**

In this retrospective autopsy study, OCM was defined as cardiac hypertrophy (≥ 400 g in men, ≥ 320 g in women) of unknown aetiology in individuals with obesity (body mass index [BMI] ≥ 25 kg/m^2^ according to the Japanese definition of obesity). We compared cases of OCM with those with obesity without cardiac hypertrophy (OB) and normal weight without cardiac hypertrophy (normal control). Macroscopically, heart weight and cardiac parameters, including epicardial adipose tissue, were measured. Fibrosis, cardiomyocyte diameter, and adipose tissue infiltration were analysed microscopically.

**Results:**

Of the 294 cases, we identified 19 cases of OCM (6.5%) and compared them with the OB and normal control groups. Patients with OCM were slightly younger than non-OCM patients (*p* = 0.081). The median heart weight was significantly heavier in OCM cases than in OB cases (435 g, interquartile range [IQR] 408–515 g vs. 360 g, IQR 341–385 g). Macroscopically, OCM hearts had a “globoid” appearance with a thickened right ventricular outflow tract. Some OCM cases showed focal interstitial fibrosis in the left ventricle. Approximately half the OCM cases were diagnosed with sudden cardiac death (SCD), with significant differences.

**Conclusions:**

The prevalence of OCM may be higher than expected in Japan, and this may be a specific pathological finding. Given that approximately half the cases of OCM were due to SCD, OCM may cause SCD, emphasizing the need to recognise and diagnose OCM.

**Supplementary Information:**

The online version contains supplementary material available at 10.1186/s12933-024-02456-z.

## Background

The prevalence of obesity is increasing worldwide, including in Japan [1–5], and the detrimental effects of obesity on cardiovascular diseases are well-established. Early observations from the eighteenth century have linked obesity, epicardial fat deposition, and sudden death [6–9]. The nineteenth century marked the first clinical report of impaired cardiac function in a patient with morbid obesity [10, 11]. This was followed by research exploring the impact of adipose tissue infiltration on heart structure and function, including ventricular hypertrophy [12], interstitial fibrosis [13], adipocyte infiltration [14], and microvascular changes [15]. These cardiac dysfunctions are referred to using various terminologies, including obesity cardiomyopathy (OCM), fatty heart, lipotoxic cardiomyopathy, cardiac steatosis, and adiposity of the heart [9, 10, 16]. The Framingham Heart Study recently solidified this link, demonstrating that obesity is an independent risk factor for heart failure and highlighting its detrimental effects on ventricular function [17]. This growing body of evidence led the American Heart Association's 2016 Scientific Statement to recognise obesity as a potential cause of cardiac dysfunction, classifying OCM as a subtype of dilated cardiomyopathy (DCM) with an endocrine or metabolic aetiology [18].

However, the question of a direct causal link between isolated obesity and human cardiomyopathy remains unaddressed. Although rodent models exhibit clear evidence of OCM [19–21], further research is needed to confirm this association in humans. Some studies have suggested a link between obesity and idiopathic DCM, whereas others have proposed a direct toxic effect of obesity on both ventricles [22–24]. Despite the expanding recognition of OCM, a standardised definition remains elusive, as OCM has several definitions, including (1) non-ischaemic DCM resulting from the direct toxic effect of lipid accumulation in myocardial cells [25, 26]; (2) myocardial disorders characterised by changes in ventricular morphology and function that occur in (morbid) obesity, including left ventricular (LV) dilation, eccentric or concentric LV hypertrophy, LV systolic and diastolic dysfunction, and right ventricular (RV) dysfunction [27]; and (3) a cardiac disorder characterised by clinically demonstrable LV dysfunction that cannot be explained by structural heart disease or systemic hypertension [28]. Among these, the British retrospective autopsy study newly defined OCM as cardiac hypertrophy without a specific cause, such as hypertension, diabetes mellitus, or valvular disease, in patients with obesity and clarified its prevalence (of 6,457 cases of sudden cardiac death [SCD], 53 [0.8%] were identified as OCM) and association with the risk of sudden death [29]. Although the prevalence of obesity is increasing in Japan, the prevalence of severe obesity (body mass index [BMI] ≥ 30 kg/m^2^) is relatively low compared with that in Western countries, and OCM is likely underestimated. However, obesity-related heart failure is becoming clinically recognised [30, 31], and clarifying the prevalence and characteristics of OCM in Japan and the possibility that OCM contributes to sudden death is crucial. Therefore, this study aimed to clarify the epidemiology and morphological/pathological features of OCM using the Japanese definition of obesity (BMI ≥ 25 kg/m^2^) and cardiac hypertrophy (men ≥ 400 g, women ≥ 320 g) based on a Japanese epidemiological study [32].

## Methods

### Study samples

This was a retrospective study conducted using data from forensic autopsy records performed at the Forensic Department, Faculty of Medicine, Oita University, from 1 January 2020 to 31 December 2023. The department is the only centre for forensic autopsies in Oita Prefecture, with approximately 150 annual autopsies performed. The Oita Prefecture is located in the Kyushu area of Japan, and the total population is approximately 1% of the Japanese population; the proportion of the population in each age group is approximately the same [33, 34]. Primary care correspondence, clinical notes, medical history, and postmortem reports, including the diagnosis of the cause of death, were reviewed to collect information on the circumstances of death and medical history. The participants did not complain of any specific symptoms to the surrounding persons and did not possess any prescription medication (including antihypertensive, antidiabetic, anticancer, and antianginal medications and non-steroidal anti-inflammatory drugs). We included only forensic autopsies, that is, those deemed necessary by the police, such as sudden death, cases of death in younger patients, or suspected incidences. Therefore, it is suitable to analyse whether OCM contributes to sudden death, as there will inevitably be more cases of sudden death. SCD in this study refers to an unexpected death occurring instantaneously or within 1 h of the development of symptoms or if unwitnessed, occurring within 24 h of last being seen well. There were no cases of primary cardiomyopathies, such as DCM or hypertrophic cardiomyopathy.

A total of 418 autopsy cases were identified. We excluded autopsy cases that showed severe postmortem changes, such as decomposition, adipocere formation, and mummification. Cases with young individuals aged < 10 years were also excluded because the weight of the heart stabilises around age 10 [32].

### Ethics statement

All procedures involving human participants were performed in accordance with the National Research Committee and the 1964 Declaration of Helsinki and its later amendments or comparable ethical standards. Our study adhered to the ethical guidelines established by the Japanese government for medical and health research involving human subjects. Ethical approval was obtained from the Ethics Committee of Oita University (reference number 2717). The requirement for informed consent was waived due to its retrospective design.

### Data collection/measurements and histopathological analysis

In Japan, the percentage of people with obesity who have a BMI of  ≥ 30 kg/m^2^ is approximately 3% for both men and women [35], which is lower than that in Europe and the United States (U.S). However, when a BMI of ≥ 25 kg/m^2^ is used to define obesity, the proportion in Japan is comparable with those in Europe and the U.S. [35]. Consistent with this, the prevalence of obesity-related diseases, such as type 2 diabetes mellitus and non-alcoholic fatty liver disease, in Japan is similar to that in Western countries [36, 37]. Additionally, the risk of developing non-alcoholic fatty liver disease in the Japanese population with BMI ≥ 25 kg/m^2^ is comparable to that in people with BMI ≥ 30 kg/m^2^ in Europe and the U.S. [35]. Therefore, in Japan, obesity is defined as excessive fat storage in adipose tissue associated with a BMI of ≥ 25 kg/m^2^, and obesity with a BMI of ≥ 35 kg/m^2^ is referred to as ‘high-degree obesity’ [35, 38]. Hence, we decided to use this definition of obesity in Japanese patients (BMI ≥ 25 kg/m^2^) in this study.

The lengths of the major and minor axes were measured in the front view of the heart (right atrial side), and (major axis-minor axis)/2 was used as roundness, with smaller values indicating a more “globoid” appearance. We also macroscopically measured the ventricular muscle wall and epicardial fat thickness along with cavity diameters at the midventricular level. The aortic, pulmonary, mitral, and tricuspid valve annuli were also measured. The right ventricular outflow tract (RVOT) was measured 10 mm below the pulmonary valve, and the left ventricular outflow tract (LOVT) was measured 10 mm below the aortic valve. Paraffin-embedded blocks were prepared for microscopic probes with sections including the left ventricle, right ventricle, coronary arteries, sinoatrial node, and atrioventricular node. The cases were categorised into groups based on BMI, which was calculated using the height and body weight measurements obtained at autopsy. Therefore, those with BMI ≥ 25 kg/m^2^ were defined as obese, and those with BMI < 24.9 kg/m^2^ were defined as normal-weight individuals. Cardiac hypertrophy of unknown aetiology was defined as a heart weight of > 400 g in males and > 320 g in females based on epidemiological data [32] in the absence of coronary artery disease (CAD), hypertension, diabetes, or valvular disease. Patients with significant CAD (obstruction > 75% of the lumen) were excluded. Infiltrating diseases, such as amyloid deposition and malignant lymphoma, were excluded from the histology. There were no cardiomyopathies, such as hypertrophic, constrictive, dilated cardiomyopathies, and arrhythmogenic right ventricular cardiomyopathy. We excluded the following aetiologies as presenting with dilated cardiomyopathy: ischaemic heart disease (including acute and chronic myocardial infarction, post-percutaneous coronary intervention state, and post-coronary artery bypass grafting state), autoimmune diseases (including rheumatoid arthritis, multiple myositis, systemic lupus erythematosus, and mixed connective tissue disease), intoxication (including alcohol, heavy metal, anticancer, non-steroidal anti-inflammatory drugs, antivirals), inflammatory conditions (including infection and sarcoidosis), infiltrating diseases (including amyloidosis and haemochromatosis), endocrinological and metabolic disorders (including thyroid dysfunction, Cushing disease, pheochromocytoma, adrenal insufficiency, growth hormone dysfunction, and diabetes mellitus), neuromuscular diseases (including muscle dystrophy and mitochondrial disease), hypertension, valvular disease, eosinophilic endocarditis, endocardial elastofibrosis, anaemia, coronary artery fistula/malformation, and arrythmia. These diagnostic criteria were then applied to individuals with obesity and normal-weight individuals. Obesity and cardiac hypertrophy of unknown aetiology were defined as OCM [29]. Two groups, the control with obesity (OB) group and the normal group, were set as follows: the OB group included individuals with a BMI ≥ 25 kg/m^2^ with a morphologically normal heart weighing < 400 g in males and 320 g in females without CAD, hypertension, diabetes, or valvular disease; the normal group included individuals with a BMI < 25.0 kg/m^2^ and a morphologically normal heart weighing < 400 g in males and 320 g in females based on a Japanese epidemiological study that revealed the heart weight of the pathological cardiac hypertrophy in Japanese population [32]. For qualitative histopathological analysis using digital images, an equal number of samples was selected from the control group to roughly match the OCM group in terms of sex and age. A total of 57 slides stained with haematoxylin and eosin (OCM, control, OB; 19 cases each) were scanned using a slide scanner (NanoZoomer-SQ, C13140-D3, Hamamatsu Photonics, Japan) to measure the cardiomyocyte diameter.

### Statistical analysis

We had no control over the sample size because of the retrospective study design; hence, no statistical sample size calculations were conducted. Categorical and binary data are presented as frequencies (percentages), and continuous data as medians and percentiles (25% and 75%). We performed quantile–quantile plot for each collected data and judged these data to be non-parametrical. The Kruskal-Wallis test and Dunn test, with Bonferroni correction if needed, were performed on non-normallydistributed or ordinal variables across the dependent group. The percentage was analysed using Fisher's exact test with Bonferroni correction if needed. The statistical software package SPSS Statistics 29 (IBM, Armonk, NY, USA) was used to perform statistical analyses. Body and cardiac parameters were also analysed with bootstrapping (by 2500 bootstrapping samples) using SPSS. The bootstrapping test for parameters was conducted with 1500 bootstrapping samples using SPSS.

## Results

### Clinical characteristics

**Fig. 1:**
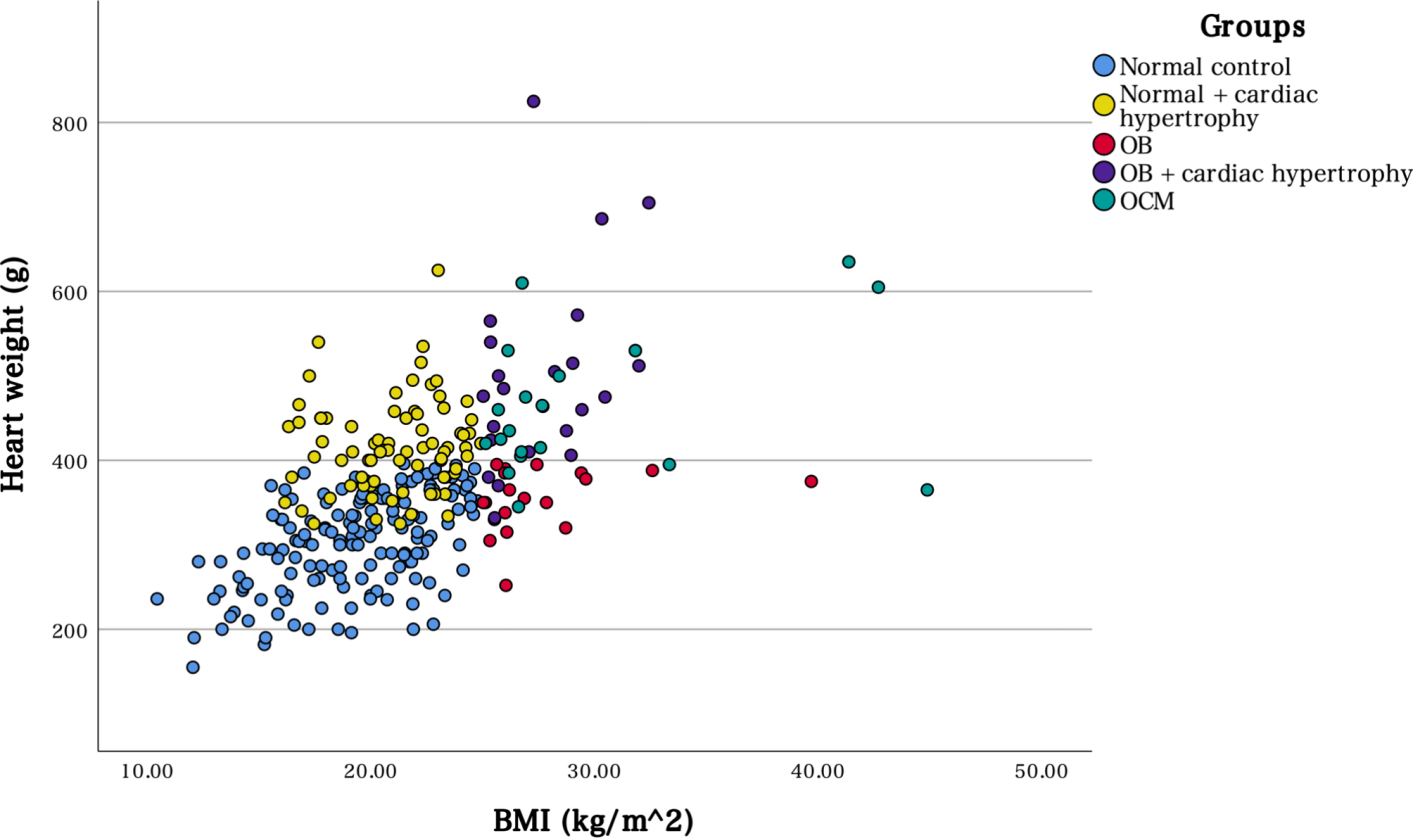
Heart weight and body mass index (BMI). Heart weight tends to increase with increasing BMI. Cardiac hypertrophy (approximately > 400 g) is associated with several aetiologies

This study included 418 participants; however, we excluded 124 participants due to postmortem changes. Among the 294 participants, we detected 19 (6%) with obesity and cardiac hypertrophy of unknown aetiology, defined as OCM (Table 1). The 19 participants with OCM were matched to 19 obese controls and 19 normal-weight controls extracted from the 294 cases. Although sex differences were not evident between the OCM and non-OCM groups (*p* = 0.447), participants in the OCM group were slightly younger than those in the non-OCM group (*p* = 0.081). Comparing the causes of death between the OCM and non-OCM groups, participants in the former group were more likely to be diagnosed with cardiovascular diseases (*p* < 0.001; odds ratio = 9.01, 95% confidence interval [CI] = 3.36–24.39). Among the OCM cases, 10 were diagnosed with SCD, and there were 15 such participants in the non-OCM group (*p* < 0.001; odds ratio = 19.23, 95% CI = 6.80–55.56; Table 1). There was a trend toward increased heart weight as the BMI increased (Fig. 1). When the Western obesity criteria (BMI ≥ 30 kg/m^2^) were used, only two patients had obesity and five had OCM.

**Table 1:** Characteristics of autopsy cases

	Overall (n = 294)	OCM (n = 19)	Non-OCM (n = 275)	OCM vs. non-OCM
	Median (25%, 75%)	Median (25%, 75%)	Median (25%, 75%)	*p*-value
Age (years)	68 (52, 78)	57 (47.5, 68)	68 (52, 78.5)	0.081
Male: female	190:104	12:7	178:97	1.000
Height (cm)	160 (152, 167)	167 (154, 172)	160 (152, 167)	0.114
BMI (kg/m^2^)	21.5 (18.3, 24.3)	26.7 (26.1, 30.1)	21.0 (18.0, 23.5)	< 0.001
Abdominal wall fat (cm)	2.0 (1.0, 2.5)	2.5 (2.3, 3.6)	2.0 (1.0, 2.5)	< 0.001
Heart weight (g)	360 (300, 410)	435 (408, 515)	355 (296, 400)	< 0.001
Cause of death				
Trauma	58 (19.7)	1 (5.3)	57 (201)	0.137
CVD	56 (19)	12 (63.2)	44 (16.0)	< 0.001
SCD	25 (8.5)	10 (52.6)	15 (5.5)	< 0.001
Ischaemic heart disease	20 (6.8)	0	20 (7.3)	0.628
Cardiac hypertrophy	7 (2.4)	1 (5.3)	6 (2.2)	0.377
Pulmonary thromboembolism	2 (0.7)	0	2 (0.7)	1.000
Cardiac sarcoidosis	1 (0.3)	0	1 (0.3)	1.000
Acute aortic dissection	1 (0.3)	1 (5.3)	0	0.065
Drowning	48 (16.3)	1 (5.3)	47 (17.1)	0.331
Asphyxial death	27 (9.2)	2 (10.5)	25 (9.1)	0.689
Poisoning	40 (13.6)	2 (10.5)	38 (13.8)	1.000
Environmental deaths	12 (4.1)	0	12 (4.4)	1.000
GI diseases	10 (3.4)	1 (5.3)	9 (3.3)	0.493
Infectious disease	10 (3.4)	0	10 (3.6)	1.000
Malnutrition	10 (3.4)	0	10 (3.6)	1.000
CNS diseases	8 (2.7)	0	8 (2.9)	1.000
Alcoholic liver disease	5 (1.7)	0	5 (1.8)	1.000
Intracranial haemorrhage	4 (1.4)	0	4 (1.5)	1.000
Malignancy	3 (1)	0	3 (1.1)	1.000
Others	3 (1)	0	3 (1.1)	1.000

BMI, body mass index; CNS, central nervous system; CVD, cardiovascular disease; SCD, sudden cardiac death; GI, gastrointestinal; OCM, obesity cardiomyopathy

Continuous data are presented as medians and percentiles (25%, 75%), and categorical data are presented as numbers and frequencies (percentages)

SCD in CVD refers to an unexpected death occurring instantaneously or within 1 h of the development of symptoms or, if unwitnessed, occurring within 24 h of last being seen well

The categories of poisoning included acute agricultural chemical toxicity, prescription drug abuse, methanol poisoning, alcohol poisoning, nicotine poisoning, hydrogen sulphide poisoning, and carbon monoxide intoxication. Carbon monoxide toxicity includes suffocation, suicide, and fire-related death; however, fire- and flame-related changes, such as burn injury, carbonisation, and thermal coagulation, were not noted. The environmental death category includes both hypothermia and heatstroke

There were increased trends in weight, BMI, or abdominal wall subcutaneous fat the OCM group compared with those in the OB group (Additional File 1 and 2), whereas heart weight was significantly higher in the OCM group than in the OB and control groups (*p* = 0.001 and *p* < 0.001, respectively) (Table 2). Compared with the normal control, the OCM group had higher heart weight, increased BMI, and more adipose tissue in the abdominal subcutaneous tissue, and this difference was significant (Table 2). At the autopsy, no other diseases were detected.

**Table 2:** Demographics and body size parameters of the three groups

	OCM (n = 19)	OB (n = 19)	*p*-value OCM vs. OB	Normal weight controls (n = 19)	*p*-value OCM vs. normal weight controls	*p*-value OB vs. normal weight controls
Age (years)	57 (47.5, 68)	50 (37.3, 61.5)	0.23	58 (47, 70.5)	1.000	0.264
Male	12	18	0.1266	12	1.000	0.127
Height (cm)	167 (154, 172)	164.5 (157.8, 168.1)	1.000	163.5 (154.75, 167)	1.000	1.000
Weight (kg)	78.0 (65.3, 83.5)	70.75 (64, 80)	1.000	50.5 (44.3, 58.3)	< 0.001	< 0.001
BMI (kg/m^2^)	26.7 (26.1, 30.1)	26.1 (26.0, 28.5)	1.000	20 (18.8, 21.8)	< 0.001	< 0.001
Abdominal wall subcutaneous fat (cm)	2.5 (2.3, 3.6)	2.5 (2.0, 3.4)	1.000	2.0 (1.5, 2.25)	0.007	0.030
Heart weight (g)	435 (408, 515)	360 (341, 385)	0.001	310 (273, 337)	< 0.001	0.099

BMI, body mass index; OB, control with obesity; OCM, obese cardiomyopathy

Continuous data are presented as medians and percentiles (25%, 75%), and categorical data are presented as numbers

### Morphological/histological characteristics

**Fig. 2:**
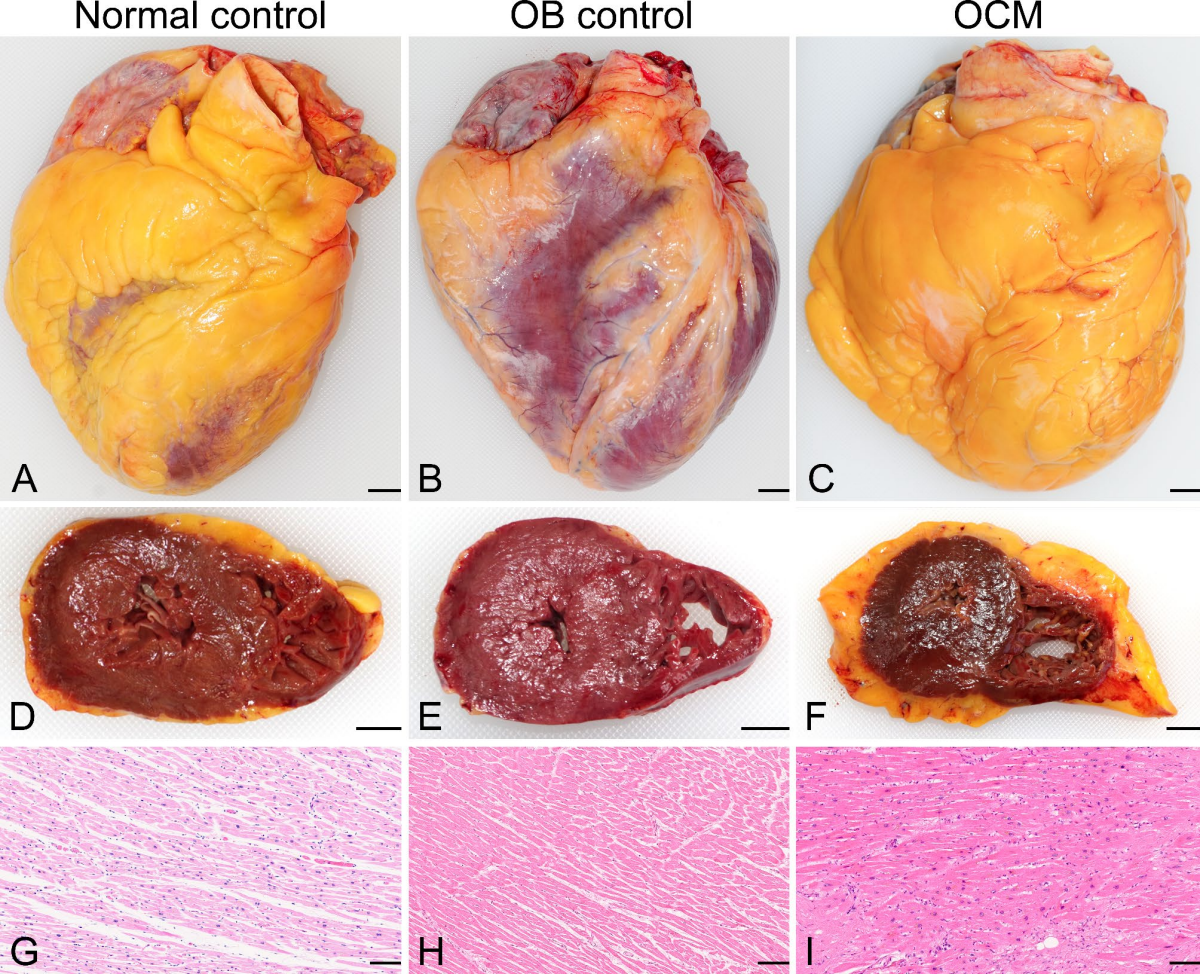
Representative pictures of each group. **A**–**F** Macroscopic view of the normal weight control (**A**), obesity control (**B**), and obesity cardiomyopathy (OCM) groups (**C**) from the anterior aspect, and the sectioning surface of the normal weight control (**D**), obesity control (**E**), and OCM groups (**F**). More severe adipose tissue deposition in the OCM group and showing “globoid” appearance” than in the other groups. **G**–**I** Haematoxylin–eosin staining of the normal weight control (**G**), obesity control (**H**), and OCM groups (**I**). The diameter of cardiomyocytes increased in the order: normal control, obesity control, and OCM groups. Adipose tissue infiltration was not significantly different among the groups. Congested capillaries with no inflammatory cell infiltration or wall thickening. Cardiomyocyte disarray is not obvious in each specimen. Scale bars indicate 1 cm (**A**–**F**) and 125 μm (**G**–**I**)

To determine whether there were morphological or histological differences, we compared the morphological/histological parameters among the three groups (Figs. 1 and 2, Table 3). Interestingly, the OCM hearts had smaller values of roundness, indicating a “globoid” appearance (Fig. 2) compared to that in the normal control group (*p* = 0.022). The mitral valve in the OCM group was longer than that in the control group. The posterior wall thickness of the LV in the OCM group was greater than that in the normal control group. More epicardial adipose tissues were deposited in the LV posterior, RV anterior, and RV lateral walls of cases in the OCM group than in the OB group, with a significant difference. The RVOT wall muscles of the OCM group were more thickened than those of the control groups (*p* = 0.002). The LVOT of the OCM group tended to be thicker than that of the OB group. Cardiomyocyte diameter was increased in the order of normal control, obese, and OCM groups, with significant differences. Contrary to expectations, there was no clear intramyocardial adipocyte infiltration in the OCM or OB group, and there were no obvious differences among the three groups. Focal fibrosis (mild) was noted in four, one, and two patients in the OCM, OB, and control groups, respectively. Replacement fibrosis (with or without hyalinosis) was not obvious in any cases. Perivascular adipocyte infiltration was evident in one patient with OCM.

**Table 3:** Demographics and cardiac pathology parameters of the three groups

	OCM (n = 19)	OB (n = 19)	*p*-value OCM vs. OB	Normal weight controls (n = 19)	*p*-value OCM vs. normal weight controls	*p*-value OB vs. normal weight controls
**Gross appearance**						
Roundness (mm) (indicator of the globoid shape)	0.25 (0.08, 0.50)	0.48 (0.21, 0.70)	0.072	0.58 (0.36, 0.71)	0.022	1.000
**Size of valves (mm)**						
Aortic valve	71.5 (70.0, 79.0)	71.0 (63.3, 75.0)	1.000	68.0 (62.5, 70.5)	0.115	0.604
Pulmonary artery valve	76.5 (70.0, 88.0)	73.5 (68.5, 84.0)	1.000	70.0 (66.0, 76.5)	0.093	0.664
Tricuspid valve	122.5 (111.5, 130.0)	121.5 (114.3, 133.8)	1.000	112.0 (106.5, 122.5)	0.204	0.210
Mitral valve	105.0 (100.3, 110.8)	105.0 (97.3, 109.0)	1.000	92.0 (90.0, 100.0)	0.007	0.015
**Left ventricle (mm)**						
Cavity diameter	20.0 (15.4, 26.1)	13.0 (10.3, 22.7)	0.329	12.0 (11.0, 13.5)	0.142	1.000
Septal wall muscle	14.0 (12.3, 15.0)	12.0 (11.0, 13.8)	0.255	12.0 (11.0, 13.5)	0.176	1.000
Anterior wall muscle	12.5 (10.6, 13.4)	10.6 (10.1, 12.4)	0.493	11.8 (10.5, 12.9)	0.981	1.000
Anterior epicardial fat	4.5 (2.9, 6.8)	3.0 (1.1, 3.2)	0.086	5.0 (2.5, 5.8)	1.000	0.423
Lateral wall muscle	14.0 (11.3, 15.0)	12.0 (11.2, 12.6)	0.343	12.0 (11.0, 14.5)	1.000	1.000
Lateral epicardial fat	2.7 (2.0, 5.1)	1.2 (0.4, 2.4)	0.245	2.8 (1.5, 3.1)	1.000	1.000
Posterior wall muscle	13.2 (12.0, 15.8)	12 (10, 13.5)	0.090	11.0 (10.0, 12.1)	0.030	1.000
Posterior epicardial fat	2.2 (1.5, 3.2)	1.1 (0.2, 2.2)	0.016	1.2 (0.4, 2.6)	0.272	0.931
LVOT wall muscle	14 (12, 16.5)	12 (11, 12.8)	0.081	12 (11, 14)	0.251	1.000
Diameter of cardiomyocyte (µm)	24.3 (22.8, 25.1)	19.2 (18.8, 19.5)	0.001	16.4 (15.8, 16.6)	< 0.001	0.001
**Right ventricle (mm)**						
Cavity diameter	17.0 (14.3, 22.5)	18.3 (14.5, 26.0)	1.000	18.0 (15.0, 22.0)	1.000	1.000
Anterior wall muscle	2.7 (2.0, 3.0)	2.2 (2.0, 3.0)	1.000	3.0 (2.2, 3.9)	0.259	1.000
Anterior epicardial fat	4.9 (3.0, 6.4)	2.2 (1.0, 3.3)	0.002	5.0 (4.1, 5.3)	1.000	0.008
Lateral wall muscle	3.0 (3.0, 4.0)	2.1 (2.0, 3.1)	1.000	2.0 (2.0, 3.1)	0.222	1.000
Lateral epicardial fat	6.3 (5.0, 8.6)	3.4 (2.8, 5.0)	0.004	4.0 (3.3, 5.3)	0.107	0.918
Posterior wall muscle	4.0 (3.5, 4.8)	3.1 (3.0, 4.0)	0.223	3.2 (3.0, 4.0)	0.530	1.000
Posterior epicardial fat	1.5 (0.6, 2.2)	0.9 (0.2, 2.6)	0.999	2.1 (0.6, 3.7)	1.000	0.267
RVOT wall muscle	3 (3, 4)	3 (2, 3)	0.406	2 (2, 3)	0.002	0.180

OCM, obesity cardiomyopathy; OB, control with obesity; LVOT, left ventricular outflow tract; RVOT, right ventricular outflow tract

Based on the bootstrapping analysis (Additional Files 1 and 2), abdominal subcutaneous fat was more thickened in the OCM group than in the normal weight control group. The aortic, pulmonary, and mitral valve annuli tended to be dilated in the OCM group compared with those in the normal weight control group. The septal and posterior wall muscles of the LV and lateral epicardial fat on the RV were more thickened in the OCM group than in the normal weight control group. The lateral and posterior wall muscles of the LV and the LVOT were more thickened in the OCM group than in the OB group. Adipose tissues were more likely to be deposited on the anterior and posterior walls of the LV and RV in the OCM group than in the OB group. The mitral valve was more dilated in the OB group than in the normal weight control group. Anterior epicardial fat deposition on the RV was more severe in the normal weight control group than in the OB group.

## Discussion

Epidemiological studies on obesity and heart failure have shown that the risk of developing heart failure has increased by 30%–100% in patients with obesity [39] and that this excess risk cannot be explained by secondary factors associated with obesity alone, such as hypertension, CAD, and diabetes; however, obesity is an independent risk factor regardless of the risk of heart failure or age and sex [31, 40–42]. The American College of Cardiology/American Heart Association guidelines also recognise obesity as an important risk factor for heart failure [41, 43]. According to Alexander, increased circulating blood volume, cardiac output, systemic hypertension, pulmonary hypertension, and decreased cardiac function can lead to heart failure [11]. This circulatory failure was thought to occur at a weight of > 135 kg, continuing over 10 years. Westaby et al. revealed the prevalence of OCM in an SCD cohort in the United Kingdom, with the definition of obesity and cardiac hypertrophy of unknown aetiology based on European standards, such as BMI and cardiac hypertrophy [29]. In the present study, we used the same definition of OCM based on the Japanese standards of BMI and cardiac hypertrophy and applied it to a set of Japanese forensic autopsy cases to clarify the prevalence of OCM, the possibility of OCM contributing to SCD, and its histopathology.

Our analysis revealed that 19 cases, approximately 6% of all cases, were categorised as OCM. Although the body weight, BMI, and heart weight values used for the definition of OCM were lower in Japan than in the European definition, the morphological features of OCM differed from those of the normal control and obesity control groups. The OCM hearts had a more “globoid” appearance than the control and OB hearts. This suggests that cardiac morphology in OCM is similar to that in DCM, which often has a “globoid” appearance. The degree of adipose tissue deposition differed between the OCM and OB groups (posterior epicardial fat on the LV and anterior/lateral epicardial fat on the RV). These results imply that the pathological adipose tissue deposition found in OCM as ectopic adipose tissue may differ from that seen in typical obesity.

Figure 1 depicts the trend toward increased heart weight as BMI increased, consistent with existing findings. However, in the absence of aetiologies such as hypertension, diabetes mellitus, and valvular diseases, the increase in heart weight was approximately 400 g, even with a high BMI. Therefore, cardiac hypertrophy of unknown aetiology is peculiar, even in patients with high BMI. This finding supports the possibility of a distinct pathophysiology of OCM independent of other obesity-related comorbidities. Based on our findings, we emphasise the existence of OCM, although body weight, BMI, and heart weight were lower than the values used in a previous British study [29].

OCM hearts showed more epicardial adipose tissue deposition, globoid shape, and thickening of the RVOT wall muscle than normal weight control hearts in our study. The characteristic haemodynamic abnormalities observed in (severely) obese individuals are increased circulating blood volume and cardiac output, resulting in congestive heart failure and dilated ventricles [11]. In obese individuals, pulmonary hypertension is believed to be caused by increased cardiac output, increased pulmonary venous pressure due to left heart failure, increased pulmonary vascular resistance due to hypoxia and other factors, and increased intrathoracic pressure [11]. Thus, the globoid shape seen in OCM hearts may be the result of haemodynamic abnormalities. Thickening of the RVOT wall muscle may reflect right heart loading due to pulmonary hypertension-like pathophysiology in OCM. Additionally, epicardial adipose tissue has been reported to contribute to myocardial dilatation and dysfunction [44]. Thus, as discussed, in addition to obesity-associated heart failure, the diastolic dysfunction induced by deposited adipose tissue may also result in congestive heart failure. Some studies reported the arrhythmogenicity of the epicardial fat [45]; therefore, fatal arrhythmia may occur in SCD cases with OCM.

In this study, wall thicknesses did not differ among the three groups, unlike in a previous study [29], although there was a trend toward myocardial wall thickening in the bootstrap sample in the order: OCM group, OB group, and control group. Increased anterior RV epicardial fat was observed in the OCM group compared to that in the other two groups. Histopathologically, mild fibrosis and adipose tissue infiltration were observed in some cases; however, significant differences were not observed, as in previous research. Considering these results, the OCM cases in our study were possibly in the early stages of OCM or were different from those in the previous study due to racial differences [29]. These results, that is, the existence of a distinct pathology that can be described as OCM, even with a lower BMI than that in the previous British study, may be related to the fact that East Asians tend to accumulate more visceral fat compared with Caucasians with similar BMIs [46, 47]. Such results have not been reported in previous research and will, therefore, help researchers and clinicians recognise undiagnosed OCM and examine it further with clinical data.

Because we used forensic autopsy cases, there was no medical bias such as age, sex, underlying disease, and comorbidities, and we could refer to cases that were more balanced than pathological autopsies. Inevitably, there were more cases of sudden death in relatively young people included in medicolegal autopsies, which makes them suitable for sudden death analysis. Remarkably, among our patients with OCM, we identified 10 cases of SCD. In this study, we did not collect SCD cases inclusively, making an accurate risk assessment of SCD difficult; however, given that approximately half the OCM cases had SCD with the odds ratio (OCM/non-OCM) of 19.23 with a 95% CI of 6.80–55.56, the possibility that OCM contributes to SCD cannot be ignored. As our cases involved prehospital deaths, the potential contribution of OCM to SCD may have been underestimated by clinicians. Furthermore, the presence of obvious OCM with a BMI of approximately 25 kg/m^2^, which may contribute to SCD, indicates that even without complications, such as hypertension or type 2 diabetes mellitus, the possibility of SCD should not be ignored, and patients could be eligible for treatment. These findings emphasise the critical need for increased awareness of the potentially life-threatening complications of OCM. Future studies will be conducted using clinical data and clinically measurable indices in patients with obesity to understand the clinical epidemiology of OCM and examine the aetiology of the disease.

Our study has some limitations. Clinical data such as echocardiography and electrocardiography and cardiac biomarkers, including atrial natriuretic peptide, brain natriuretic peptide, and N-terminal pro-brain natriuretic peptide, are lacking. Although morphologically clear differences were pointed out among the OCM, OB, and control groups, the clinical presentations were unclear. In future studies, we hope to integrate OCM with more severe obesity and clinical data to provide clinical significance. Histopathological changes are important for understanding the underlying mechanisms that may lead to SCD or heart failure, and we hope to incorporate this information in future studies.

Regarding future clinical application, increased abdominal subcutaneous fat and adipose tissue deposition on the heart may distinguish patients with OCM from those with pure obesity based on the results. A prospective study focusing on this point, with echocardiographic measurements and serological assessments, may make it possible to distinguish OCM from simple obesity. For cases of obesity-associated heart failure or cases thought to be OCM, weight loss, including bariatric weight-loss surgery, has been reported to improve symptoms [48–50]. Therefore, appropriate diagnosis and therapeutic intervention may help prevent heart failure and SCD. We are also considering comparisons with other ethnicities to obtain more universal results in subsequent studies.

## Conclusions

Approximately 6% of the participants in this study were classified as having OCM with cardiac hypertrophy of unknown aetiology. In these cases, the microscopic differences were nonspecific and characterised primarily by adipose tissue deposits on the epicardial side. A “globoid” appearance and adipose tissue deposition deferred from typical obesity imply a distinguishing pathology such as DCM. This could contribute to SCD, and further clinical research is needed.

## Electronic supplementary material

Supplementary Material 1

Supplementary Material 2

## Data Availability

The datasets obtained and analysed in the current study are available from the corresponding author upon reasonable request.

## Abbreviations

OCM: Obesity cardiomyopathy
BMI: Body mass index
SCD: Sudden cardiac death
DCM: Dilated cardiomyopathy
LV: Left ventricular
RV: Rventricular
U.S.: United States of America
CAD: Coronary artery diseases
