# Supplementary material for: Obesity cardiomyopathy could contribute to sudden cardiac death: a Japanese epidemiological morphological study
Source: Cardiovasc Diabetol. 2024 Oct 24;23:378. doi: 10.1186/s12933-024-02456-z (PMC11520142; doi:10.1186/s12933-024-02456-z)
Supplement: Supplementary file 1 — Supplementary Material 1 [file 12933_2024_2456_MOESM1_ESM.docx]

**Additional File 1. Cardiac parameters with bootstrapping data (2500 samples).**

|  | OCM | | OB | | Normal weight controls | |
| --- | --- | --- | --- | --- | --- | --- |
|  | Mean | 95% CI | Mean | 95% CI | Mean | 95% CI |
| Age (years) | 58.11 | 49.98, 66.24 | 50.06 | 40.21, 59.92 | 57.00 | 47.47, 66.53 |
| Height (cm) | 164.14 | 159.29, 169.00 | 163.44 | 159.37, 167.50 | 162.13 | 155.58, 168.67 |
| Weight (kg) | 81.19 | 71.02, 91.37 | 72.25 | 66.92, 77.58 | 53.83 | 47.48, 60.18 |
| BMI (kg/m^2^) | 29.97 | 26.81, 33.13 | 26.94 | 25.82, 28.06 | 20.31 | 19.07, 21.55 |
| Abdominal wall subcutaneous fat (cm) | 2.99 | 2.28, 3.70 | 2.79 | 2.02, 3.56 | 1.99 | 1.62, 2.35 |
| Heart weight (g) | 455.56 | 416.28, 494.83 | 355.06 | 334.09, 376.04 | 312.73 | 287.73, 337.73 |
| **Size of valves (mm)** |  |  |  |  |  |  |
| Aortic valve | 72.17 | 67.76, 76.57 | 70.25 | 65.17, 75.33 | 66.07 | 62.43, 69.71 |
| Pulmonary artery valve | 78.61 | 73.21, 84.02 | 75.88 | 70.36, 81.39 | 70.60 | 66.64, 74.56 |
| Tricuspid valve | 118.17 | 104.05, 132.29 | 123.50 | 113.86, 133.14 | 115.00 | 108.09, 121.91 |
| Mitral valve | 105.28 | 100.59, 109.96 | 103.93 | 97.58, 110.29 | 94.33 | 90.23, 98.44 |
| **Left ventricle (mm)** |  |  |  |  |  |  |
| Cavity diameter | 20.26 | 16.31, 24.21 | 15.68 | 11.91, 19.45 | 14.95 | 11.11, 18.78 |
| Septal wall muscle | 14.06 | 12.46, 15.65 | 12.53 | 11.78, 13.27 | 12.26 | 11.16, 13.36 |
| Anterior wall muscle | 12.13 | 11.11, 13.14 | 17.83 | 3.98, 31.67 | 11.48 | 10.46, 12.50 |
| Anterior epicardial fat | 4.94 | 3.67, 6.21 | 2.94 | 1.71, 4.17 | 4.16 | 2.99, 5.33 |
| Lateral wall muscle | 13.69 | 11.99, 15.39 | 10.81 | 8.54, 13.08 | 12.63 | 11.46, 13.81 |
| Lateral epicardial fat | 3.81 | 2.11, 5.50 | 2.35 | 0.89, 3.81 | 2.69 | 1.61, 3.78 |
| Posterior wall muscle | 13.64 | 12.35, 14.94 | 11.04 | 8.96, 13.13 | 10.71 | 8.86, 12.56 |
| Posterior epicardial fat | 2.60 | 1.69, 3.51 | 1.14 | 0.53, 1.75 | 1.60 | 0.87, 2.33 |
| LVOT wall muscle | 14.17 | 12.53, 15.80 | 11.81 | 10.92, 12.71 | 12.40 | 11.22, 13.58 |
| Diameter of cardiomyocyte (μm) | 24.46 | 23.69, 25.24 | 19.09 | 18.83, 19.36 | 15.92 | 15.40, 16.45 |
| **Right ventricle (mm)** |  |  |  |  |  |  |
| Cavity diameter | 18.79 | 15.59, 22.00 | 19.46 | 15.17, 23.75 | 18.55 | 15.25, 21.84 |
| Anterior wall muscle | 2.53 | 2.29, 2.77 | 2.37 | 1.87, 2.87 | 3.00 | 2.46, 3.54 |
| Anterior epicardial fat | 5.19 | 3.72, 6.67 | 2.07 | 1.26, 2.88 | 4.47 | 3.25, 5.69 |
| Lateral wall muscle | 3.34 | 2.77, 3.92 | 2.91 | 2.27, 3.56 | 2.53 | 1.83, 3.24 |
| Lateral epicardial fat | 7.27 | 5.06, 9.48 | 3.68 | 2.67, 4.68 | 4.57 | 3.29, 5.85 |
| Posterior wall muscle | 4.11 | 3.49, 4.73 | 3.34 | 2.78, 3.91 | 3.59 | 2.85, 4.33 |
| Posterior epicardial fat | 1.67 | 1.02, 2.31 | 1.29 | 0.60, 1.98 | 2.33 | 1.32, 3.33 |
| RVOT wall muscle | 3.44 | 2.90, 3.99 | 2.88 | 2.33, 3.42 | 2.20 | 1.72, 2.68 |

OCM, obesity cardiomyopathy; OB, control with obesity; LVOT, left ventricular outflow tract; RVOT, right ventricular outflow tract; CI, confidential interval
