# Supplementary material for: Obesity cardiomyopathy could contribute to sudden cardiac death: a Japanese epidemiological morphological study
Source: Cardiovasc Diabetol. 2024 Oct 24;23:378. doi: 10.1186/s12933-024-02456-z (PMC11520142; doi:10.1186/s12933-024-02456-z)
Supplement: Supplementary file 2 — Supplementary Material 2 [file 12933_2024_2456_MOESM2_ESM.docx]

**Additional File 2. Bootstrap test for cardiac parameters (1500 bootstrapping samples).**

|  | OCM vs. OB | | | | | | | OCM vs. Normal weight controls | | | | | | | OB vs. Normal weight controls | | | | | | |
| --- | --- | --- | --- | --- | --- | --- | --- | --- | --- | --- | --- | --- | --- | --- | --- | --- | --- | --- | --- | --- | --- |
|  |  |  | 95% CI of MD | |  | 95% CI of d | |  |  | 95% CI of MD | |  | 95% CI of d | |  |  | 95% CI of MD | |  | 95% CI of d | |
|  | MD | p | Lower | Upper | d | Lower | Upper | MD | p | Lower | Upper | d | Lower | Upper | MD | p | Lower | Upper | d | Lower | Upper |
| Age (years) | -8.05 | 0.1905 | -20.00 | 3.43 | -0.46 | -1.14 | 0.22 | -1.11 | 0.8541 | -13.74 | 10.27 | -0.07 | -0.75 | 0.62 | 6.94 | 0.2738 | -4.85 | 19.21 | 0.39 | -0.33 | 1.10 |
| Height (cm) | -0.71 | 0.8108 | -6.40 | 5.40 | -0.08 | -0.75 | 0.59 | -2.02 | 0.6076 | -9.16 | 5.62 | -0.19 | -0.87 | 0.50 | -1.31 | 0.7169 | -8.53 | 5.70 | -0.13 | -0.84 | 0.57 |
| Weight (kg) | -8.94 | 0.1226 | -19.62 | 1.16 | -0.55 | -1.23 | 0.15 | -27.36 | 0.0013 | -39.30 | -15.99 | -1.61 | -2.39 | -0.81 | -18.42 | 0.0007 | -26.17 | -10.52 | -1.72 | -2.54 | -0.87 |
| BMI (kg/m^2^) | -3.03 | 0.1033 | -6.44 | -0.08 | -0.62 | -1.31 | 0.07 | -9.66 | 0.0047 | -13.32 | -6.57 | -1.95 | -2.78 | -1.10 | -6.63 | 0.0007 | -8.08 | -5.18 | -3.06 | -4.10 | -2.00 |
| Abdominal wall subcutaneous fat (cm) | -0.20 | 1.0000 | -1.08 | 0.80 | -0.14 | -0.81 | 0.54 | -1.00 | 0.0173 | -1.78 | -0.27 | -0.87 | -1.59 | -0.15 | -0.80 | 0.0886 | -1.60 | -0.06 | -0.70 | -1.43 | 0.03 |
| Heart weight (g) | -100.49 | 0.0020 | -143.34 | -60.83 | -1.58 | -2.35 | -0.80 | -142.82 | 0.0007 | -187.09 | -99.82 | -2.17 | -3.03 | -1.29 | -42.33 | 0.0127 | -71.89 | -13.37 | -1.00 | -1.74 | -0.24 |
| **Size of valves (mm)** |  |  |  |  |  |  |  |  |  |  |  |  |  |  |  |  |  |  |  |  |  |
| Aortic valve | -0.19 | 1.0000 | -0.83 | 0.40 | -0.21 | -0.88 | 0.47 | -0.61 | 0.0393 | -1.13 | -0.08 | -0.77 | -1.48 | -0.05 | -0.42 | 0.1626 | -0.98 | 0.18 | -0.51 | -1.22 | 0.21 |
| Pulmonary artery valve | -0.27 | 1.0000 | -1.02 | 0.42 | -0.26 | -0.93 | 0.42 | -0.80 | 0.0220 | -1.40 | -0.19 | -0.85 | -1.57 | -0.13 | -0.53 | 1.0000 | -1.12 | 0.10 | -0.59 | -1.30 | 0.14 |
| Tricuspid valve | 0.53 | 0.5563 | -0.89 | 2.19 | 0.22 | -0.46 | 0.89 | -0.32 | 0.6889 | -1.56 | 1.40 | -0.14 | -0.82 | 0.55 | -0.85 | 0.1233 | -1.88 | 0.25 | -0.54 | -1.26 | 0.18 |
| Mitral valve | -0.13 | 1.0000 | -0.82 | 0.62 | -0.13 | -0.80 | 0.55 | -1.09 | 0.0027 | -1.66 | -0.53 | -1.28 | -2.02 | -0.51 | -0.96 | 0.0120 | -1.71 | -0.26 | -0.96 | -1.70 | -0.21 |
| **Left ventricle (mm)** |  |  |  |  |  |  |  |  |  |  |  |  |  |  |  |  |  |  |  |  |  |
| Cavity diameter | -0.46 | 0.0933 | -0.96 | 0.06 | -0.61 | -1.29 | 0.09 | -0.53 | 0.0580 | -1.04 | -0.03 | -0.71 | -1.41 | 0.00 | -0.07 | 1.0000 | -0.54 | 0.42 | -0.10 | -0.81 | 0.60 |
| Septal wall muscle | -0.14 | 1.0000 | -0.31 | 0.02 | -0.55 | -1.23 | 0.14 | -0.19 | 0.0513 | -0.39 | -0.01 | -0.67 | -1.37 | 0.04 | -0.05 | 1.0000 | -0.19 | 0.10 | -0.24 | -0.94 | 0.47 |
| Anterior wall muscle | 0.57 | 0.4637 | -0.19 | 2.09 | 0.32 | -0.36 | 0.99 | -0.06 | 1.0000 | -0.20 | 0.06 | -0.33 | -1.02 | 0.36 | -0.63 | 1.0000 | -2.18 | 0.12 | -0.34 | -1.05 | 0.37 |
| Anterior epicardial fat | -0.20 | 0.0286 | -0.37 | -0.02 | -0.82 | -1.52 | -0.11 | -0.08 | 1.0000 | -0.24 | 0.07 | -0.33 | -1.02 | 0.36 | 0.12 | 0.1452 | -0.04 | 0.27 | 0.55 | -0.17 | 1.26 |
| Lateral wall muscle | -0.29 | 0.0593 | -0.56 | -0.04 | -0.75 | -1.44 | -0.05 | -0.11 | 0.3005 | -0.30 | 0.08 | -0.36 | -1.05 | 0.33 | 0.18 | 0.1526 | -0.02 | 0.42 | 0.54 | -0.19 | 1.25 |
| Lateral epicardial fat | -0.15 | 0.2012 | -0.37 | 0.07 | -0.47 | -1.15 | 0.22 | -0.11 | 0.2712 | -0.31 | 0.05 | -0.39 | -1.08 | 0.30 | 0.03 | 0.6795 | -0.14 | 0.20 | 0.14 | -0.56 | 0.85 |
| Posterior wall muscle | -0.26 | 0.0366 | -0.49 | -0.04 | -0.79 | -1.49 | -0.09 | -0.29 | 0.0213 | -0.50 | -0.09 | -0.99 | -1.71 | -0.26 | -0.03 | 0.7968 | -0.29 | 0.22 | -0.09 | -0.80 | 0.61 |
| Posterior epicardial fat | -0.15 | 0.0153 | -0.25 | -0.06 | -0.95 | -1.65 | -0.23 | -0.10 | 0.0740 | -0.21 | 0.00 | -0.62 | -1.32 | 0.09 | 0.05 | 1.0000 | -0.04 | 0.14 | 0.38 | -0.34 | 1.08 |
| LVOT wall muscle | -0.24 | 0.0127 | -0.40 | -0.06 | -0.88 | -1.58 | -0.17 | -0.18 | 0.0786 | -0.36 | 0.02 | -0.62 | -1.32 | 0.08 | 0.06 | 0.3877 | -0.07 | 0.19 | 0.31 | -0.40 | 1.01 |
| Diameter of cardiomyocyte (μm) | -5.37 | 0.0007 | -6.13 | -4.62 | -4.51 | -5.79 | -3.21 | -8.54 | 0.0007 | -9.43 | -7.73 | -6.45 | -8.18 | -4.71 | -3.17 | 0.0007 | -3.76 | -2.68 | -4.23 | -5.51 | -2.92 |
| **Right ventricle (mm)** |  |  |  |  |  |  |  |  |  |  |  |  |  |  |  |  |  |  |  |  |  |
| Cavity diameter | 0.07 | 0.7855 | -0.40 | 0.56 | 0.09 | -0.58 | 0.77 | -0.02 | 0.9174 | -0.43 | 0.40 | -0.04 | -0.72 | 0.65 | -0.09 | 0.7348 | -0.59 | 0.42 | -0.13 | -0.83 | 0.58 |
| Anterior wall muscle | -0.02 | 1.0000 | -0.06 | 0.04 | -0.22 | -0.89 | 0.46 | 0.05 | 1.0000 | -0.01 | 0.10 | 0.63 | -0.07 | 1.33 | 0.06 | 1.0000 | -0.01 | 0.13 | 0.66 | -0.07 | 1.38 |
| Anterior epicardial fat | -0.31 | 0.0033 | -0.48 | -0.15 | -1.30 | -2.04 | -0.55 | -0.07 | 0.4404 | -0.26 | 0.09 | -0.27 | -0.96 | 0.42 | 0.24 | 0.0020 | 0.11 | 0.37 | 1.28 | 0.49 | 2.05 |
| Lateral wall muscle | -0.04 | 0.3005 | -0.12 | 0.04 | -0.37 | -1.04 | 0.32 | -0.08 | 1.0000 | -0.16 | 0.00 | -0.67 | -1.37 | 0.04 | -0.04 | 1.0000 | -0.12 | 0.04 | -0.31 | -1.01 | 0.41 |
| Lateral epicardial fat | -0.36 | 0.0167 | -0.61 | -0.16 | -1.03 | -1.74 | -0.30 | -0.27 | 0.0646 | -0.54 | -0.05 | -0.74 | -1.45 | -0.03 | 0.09 | 0.2458 | -0.05 | 0.24 | 0.42 | -0.29 | 1.13 |
| Posterior wall muscle | -0.08 | 0.0746 | -0.16 | 0.00 | -0.66 | -1.35 | 0.04 | -0.05 | 0.2725 | -0.14 | 0.03 | -0.41 | -1.10 | 0.29 | 0.02 | 0.5949 | -0.06 | 0.10 | 0.20 | -0.51 | 0.91 |
| Posterior epicardial fat | -0.04 | 0.4151 | -0.12 | 0.04 | -0.29 | -0.96 | 0.39 | 0.07 | 0.2678 | -0.05 | 0.17 | 0.43 | -0.27 | 1.12 | 0.10 | 0.0873 | -0.01 | 0.21 | 0.66 | -0.07 | 1.38 |
| RVOT wall muscle | -0.06 | 0.1326 | -0.13 | 0.02 | -0.54 | -1.22 | 0.15 | -0.12 | 0.0007 | -0.19 | -0.06 | -1.25 | -1.99 | -0.49 | -0.07 | 1.0000 | -0.13 | 0.00 | -0.71 | -1.43 | 0.02 |

OCM, obesity cardiomyopathy; OB, control with obesity; LVOT, left ventricular outflow tract; RVOT, right ventricular outflow tract; MD, mean difference; p, two-tailed p- value; d, Cohen’s d effect size; CI, confidential interval.

The two-tailed p-values shown in this table are not corrected by Bonferroni’s correction.
